# Peanut Sensitization Profiles in Italian Children and Adolescents with Specific IgE to Peanuts

**DOI:** 10.1155/2013/170452

**Published:** 2013-11-14

**Authors:** Elisabetta Calamelli, Carlo Caffarelli, Giampaolo Ricci

**Affiliations:** ^1^UO Pediatria, Dipartimento di Scienze Mediche e Chirurgiche, University of Bologna, Via Massarenti 11, 40138 Bologna, Italy; ^2^Pediatric Department, Azienda Ospedaliera-Universitaria, Department of Clinical and Experimental Medicine, University of Parma, Via Gramsci 14, 43126 Parma, Italy

## Abstract

Peanuts are one of the most relevant foods implicated in IgE-mediated adverse reactions in pediatric population. This study aimed to evaluate the pattern of sensitization towards five peanut allergenic components (rAra h 1, 2, 3, 8 and 9) in a population of Italian children and adolescents with specific IgE (sIgE) to peanut. rAra h 9 was the main allergen implicated in peanut sensitization (58%), followed by rAra h 8 (35%), rAra h 2 (27%), rAra h 3 (23%) and rAra h 1 (12.5%). rAra h 1, 2, and 3 were the main allergenic components in young children: 8/13 (62%) between 2 and 5 years, 8/23 (35%) between 6 and 11 years, and 3/12 (25%) between 1 and 16 years. No differences were found among the levels of sIgE towards rAra h 1, 2, 3, and 9 in the three groups; in contrast, the levels of sIgE against rAra h 8 showed an increasing trend according to age. In conclusion rAra h 1, 2, and 3 were the prevalent sensitizing allergens during the first years of life in Italian patients with sIgE to peanuts (“genuine” allergy); in contrast rAra h 9 and 8 were mainly involved in school-age children and adolescents with pollen allergy (“secondary” sensitization).

## 1. Introduction

Peanuts (*Arachis hypogaea*) are one of the most relevant foods implicated in IgE-mediated adverse reactions, due to the frequency and severity of clinical manifestations [1]. Peanut allergy is estimated to affect from 0.5 up to 1.8% of children in western countries [2–7]. In the last decade, the component-resolved diagnosis (CRD) approach has allowed the identification of specific IgE (sIgE) to different allergenic components of peanuts [8, 9]. Until now, thirteen peanuts allergens have been recognized (Ara h 1–13) [10]. Among them, the seed storage proteins (SSPs) Ara h 1, Ara h 2 and Ara h 3 are the major peanut allergens and are considered to be the markers of genuine IgE-mediated peanut allergy [11, 12]. On the other hand, Ara h 8, a Bet v 1 homologue, is a pathogenesis-related protein 10 (PR-10) implicated in mechanisms of cross sensitization between pollens and foods [13], and the nonspecific lipid transfer protein (nsLTP) Ara h 9 is an allergen prevalent in the Mediterranean area [14]. The sensitization against different allergenic components can help to predict the possible severity of symptoms [15]. Indeed, the SSPs allergens are heat stable and resistant to enzymatic digestion and are implicated in systemic and life-threatening reactions [16, 17]. Ara h 8 is considered a marker of cross-reactivity between inhalant and food allergens, is rapidly digested by gastric enzymes, and is implicated in mild local reactions, such as oral allergy syndrome (OAS) [13, 18]. The LTPs are relevant allergens in patients from the Mediterranean area and are often implicated not only in local symptoms, but also in severe systemic reactions [14]. This study aimed to evaluate the pattern of specific IgE (sIgE) against these five main allergenic components of *Arachis hypogaea* in a population of Italian children and adolescents with documented peanut sensitization (sIgE > 1 kUA/L) and a clinical history of suspected peanut and/or tree nuts allergy.

## 2. Materials and Methods

### 2.1. Study Population

The study involved 48 children (30 males, 62%, and 18 females, 38%) with a median age of 8 years (range 2–16 years) referred to the Pediatric Allergology Unit of Bologna University from November 2011 until December 2012. Inclusion criteria were a clinical history of suspected peanut and/or tree nuts allergy and a level of sIgE against peanut ≥ 1 kUA/L (ImmunoCAP 1000 Thermo Fisher Scientific, Uppsala, Sweden). Symptoms suggestive of atopic dermatitis and food and or respiratory allergy (asthma and rhino-conjunctivitis) were investigated in all enrolled patients. Parental history of atopy and the age of onset of allergic diseases were asked for all patients and/or to their parents. Of the forty-eight patients recruited for this study, 24 (50%) followed an elimination diet for peanuts or had never eaten them and were classified as “avoiders.” The rest of them, who used to eat peanuts without adverse reactions were defined to be “tolerant.” This research was performed in accordance with the principles of the Declaration of Helsinki. 

### 2.2. Determination of Specific IgE

Serum sIgE was detected by the means of the CAP system (ImmunoCAP 1000 Thermo Fisher Scientific, Uppsala, Sweden). The determination of sIgE against a panel of inhalant [pollen from grass (*Phleum pratense* and *Cynodon dactylon*), birch (*Betula verucosa*), hazel (*Corylus avellana*) and olive tree (*Olea europea*)] and food allergens (peanut and its components (rAra h 1, rAra h 2, rAra h 3, r Ara h 8, and rAra h 9), hazelnut, walnut, rice, wheat, soy and apple) was performed in all patients' sera. Levels of serum sIgE greater than 0.35 kUA/L were considered positive.

### 2.3. Statistical Methods

Data were stored by means of customized databases. Statistical analyses were carried out by means of MedCalc statistical software (Version 12.5.0, MedCalc Software, Ostend, Belgium). The Chi-square test and nonparametrical tests were applied when appropriate. In particular, proportions were compared by Chi-square test; geometric mean levels of sIgE were compared by Mann-Whitney *U*-test. Probability (*P*) values of less than 0.05 were considered significant.

## 3. Results and Discussion

### 3.1. Results

The clinical and serological characteristics of the study population are resumed in Table 1. rAra h 9 was the main allergen implicated in peanut sensitization (58%), followed by rAra h 8 (35%), rAra h 2 (27%), rAra h 3 (23%) and rAra h 1 (12.5%). Twenty-six patients (54%) were sensitized to only one of the five allergenic components and in two cases (4%) none of the five investigated allergens were implicated. Twenty-four out of the 48 patients (50%) used to avoid peanuts' consumption and were defined as “avoiders.” Detailed clinical and laboratory features of the study population categorized in the two subgroups (tolerant/avoiders) are resumed in Table 1. Among the group avoiding peanuts, 20 had never eaten peanuts; on the other hand four of them had experienced adverse reactions after peanuts ingestion (two cases of anaphylaxis, one of generalized urticaria and in one case oral allergy syndrome). Moreover, patients avoiding peanuts showed an higher rate of sensitization towards rAra h 1 and rAra h 2 than the tolerant group (resp. 25% versus 0% for rAra h 1 and 42% versus 12% for rAra h 2; *P* < 0.05). Furthermore, patients avoiding peanuts showed a higher level of sIgE against peanut extract (12.5 kUA/L versus 6.5 kUA/L) and rAra h 2 (7.5 kUA/L versus 2 kUA/L) than the tolerant ones (*P* < 0.05). As shown in Table 2, half of the children avoiding peanuts were sensitized to more than one peanut component, compared to the 33% of tolerant patients (not significant).

**Figure 1:**
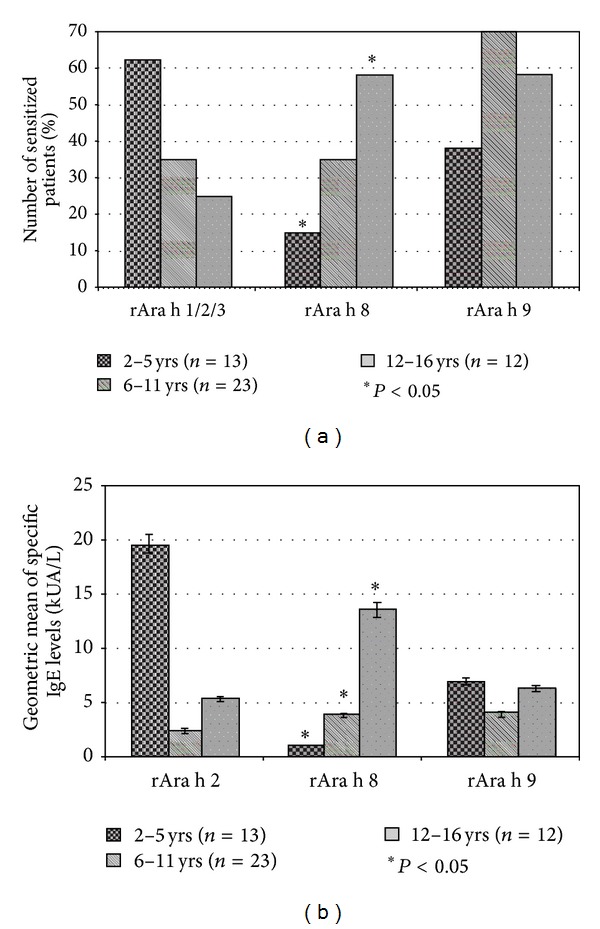
Peanut specific IgE profiles of the 48 children and adolescents (median age: 8 years; range: 2–16) with documented peanut sensitization (sIgE > 1 kUA/L). Patients have been grouped in three age categories (2–5 years old (*n* = 13), 6–11 years old (*n* = 23), and 12–16 years old (*n* = 12)). (a) Rates of sensitization against one of the five peanuts' molecular components rAra h 1, 2, and 3 (seed storage proteins), rAra h 8 (pathogenesis-related protein 10) and rAra h 9 (lipid transfer protein) in the study population. The rate of sensitization against the seed storage proteins rAra h 1, 2, and 3 is referred to the positivity (specific IgE > 0.35 kUA/L) to at least one of the three above-mentioned allergens. (b) Geometric means (error bars: 95% confidence intervals) of specific IgE against rAra h 2 (the most frequent seed storage Protein in our patients), rAra h 8 and 9 in the study population. Probability (*P*) values of less than 0.05 were considered significant.

We grouped patients in three age categories (2–5 years old (*n* = 13), 6–11 years old (*n* = 23) and 12–16 years old (*n* = 12)) to evaluate differences in their molecular profiles (Figure 1). The SSPs were the main allergenic components implicated in peanut sensitization during the preschool age: 8/13 (62%) between 2–5 years, 8/23 (35%) between 6 and 11 years and only 3 patients out of 12 (25%) between 12 and 16 years. In contrast, the Bet v 1 homologue rAra h 8 and the LTP rAra h 9 were prevalent in school children and adolescents. The analysis of the geometric means of sIgE levels showed no relevant differences in the three age groups concerning both the SSPs and the LTPs, whereas the levels of sIgE against rAra h 8 showed an increasing trend according to age (from 1 kUA/L in preschool children to 4 kUA/L until 13.5 kUA/L in adolescents).

### 3.2. Discussion

Our results confirm the relevance of the LTP Ara h 9 (positive in 58% of our study population) as a major allergen in peanut sensitized subjects living in a country of the Mediterranean area [14]. Comparable results were shown by Vereda et al., who investigated the sensitization profiles of Spanish children and adolescents with peanut allergy [19]. Meanwhile, Spanish patients showed a lower percentage of sensitization towards Ara h 8 (only 2% versus 35% in our study population) and Bet v 1 (6.5% versus 37% among our patients). These data reflect the lower birch pollen exposure among Spanish children and adolescents, compared to subjects from our geographical region (north of Italy). Indeed, the vegetation and plant distribution across the European countries shows relevant geographical differences from the northern to the southern regions, with a strong impact on the sensitization profiles of the living population [20]. As shown from the Italian observational survey “Pan-allergens in Pediatrics,” children living in the Northern Italy have peculiar pattern of sensitization against plant-derived allergens (e.g., grasses, birch, hazel tree, and peanut) which differs from the population of the South of the peninsula [21]. Interestingly, although in Italy the population mean of daily intake of peanuts is low (0.05 g/day versus 0.19 g/day in Europe), we found a relevant percentage of patients with peanut genuine sensitization (40% of our patients are sensitized to at least one of the SSPs), and most of them were small children [22]. Unfortunately, no data are available about the peanuts' daily intake in our study population, nor about the maternal consumption during pregnancy. Half of our children avoiding peanuts were sensitized to more than one peanut component, compared to the 33% of tolerant patients. Despite the fact that no significant differences were found between the two groups, our data are supported by previous researches, which highlighted more severe reactions in peanut allergic patients recognizing a greater number of allergens [23, 24].

Interestingly, our data showed an age-dependent sensitization pattern in patients with sIgE against peanut extract. Pre-school children were mostly sensitized (62%) to one of the SSPs peanuts allergens. In contrast, the Bet v 1 homologue Ara h 8 and the LTP Ara h 9 were prevalent in school children and adolescents, with levels of sIgE against Ara h 8 increasing with age. Since the sensitization to one of the peanuts' SSPs (in particular Ara h 2) has been proved to be a marker of positive oral provocation test in patients with suspected peanut allergy, our findings show a possible more relevant risk of clinical reactions in patients with early onset of peanut sensitization [25]. 

The methodological limitations of this research need to be mentioned. Indeed, although the component-resolved diagnosis approach showed a detailed picture of the molecules implicated in the mechanism of peanut sensitization, the lack of complementary diagnostic tests (e.g., skin prick test, oral provocation test, and metabolite evaluation) is a bias of this study [26]. In particular, the oral provocation test performed with peanuts might have better ascertained the tolerance status of those patients who had never eaten peanuts. 

Despite these limitations, our data highlight the need for further research focusing on this phenomenon. 

## 4. Conclusions

In conclusion, in our study the LTP rAra h 9 was the main implicated allergen in Italian patients with sIgE to peanuts, with similar prevalence in tolerant patients and patients avoiding peanuts. Moreover, both rAra h 9 and rAra h 8 were mainly involved in peanut sensitization in school age children and adolescents, with sIgE levels increasing with age, probably due to mechanism of cross-reactivity (secondary sensitization) in patients with pollen allergy. In contrast, the SSPs, in particular Ara h 1 and Ara h 2, were the prevalent sensitizing allergenic components in peanut avoiders, and their rates of sensitization were higher in preschool children, showing a phenomenon of genuine IgE-specific sensitization. Further research is needed to prove the clinical implications of these data. 

## Figures and Tables

**Table 1:** Clinical and laboratory features of the 48 children and adolescents with documented peanut sensitization (sIgE > 1 kUA/L). The patients have been categorized by the tolerance status against peanuts.

	Study population (*n* = 48)	Tolerant patients (*n* = 24)	Avoiders (*n* = 24)	*P* values Tolerant versus avoiders
Sex, male, *n* (%)	30 (62)	12 (50)	18 (75)	NS
Age, median years (range)	8 (2–16)	9.5 (2–16)	8 (2–16)	NS
Parental history of atopy, *n* (%)	21 (44)	8 (33)	13 (54)	NS
Allergic respiratory disease, *n* (%)	26 (54)	13 (54)	13 (54)	NS
Age onset respiratory disease years, median (range)	6 (2–13)	6.5 (4–10)	4.5 (2–13)	NS
Atopic Dermatitis, *n* (%)	27 (56)	11 (46)	16 (67)	NS
Age onset atopic dermatitis years, median (range)	0.5 (0.5–5)	0.8 (0.5–4)	0.5 (0.5–5)	NS
Food allergy, *n* (%)	40 (83)	16 (67)	24 (100)	<0.005
Age onset food allergy years, median (range)	1 (0.5–11)	1 (0.5–6)	1.5 (0.5–11)	NS
Peanut avoiders, *n* (%)	24 (50)	0	24 (100)	NA
Never eaten, *n* (%)	20 (42)	0	20 (83)	NA
Clinical reactions, *n* (%)	4 (8)	0	4 (17)	NA
Inhalant allergen sensitization, *n* (%)	40 (83)	20 (83)	20 (83)	NS
Grass (Phleum p. and/or Cynodon d.), *n* (%)	39 (81)	19 (79)	20 (83)	NS
Birch, *n* (%)	36 (75)	17 (71)	19 (79)	NS
Bet v 1, *n* (%)	18 (37)	7 (29)	9 (37)	NS
Hazel tree, *n* (%)	36 (75)	18 (75)	18 (75)	NS
Olive tree, *n* (%)	37 (77)	18 (75)	19 (79)	NS
Food allergen sensitization, *n* (%)	48 (100)	24 (100)	24 (100)	NA
Peanut, *n* (%)	48 (100)	24 (100)	24 (100)	NA
Ara h 1, *n* (%)	6 (12.5)	0 (0)	6 (25)	<0.05
Ara h 2, *n* (%)	13 (27)	3 (12)	10 (42)	<0.05
Ara h 3, *n* (%)	11 (23)	4 (17)	7 (29)	NS
Ara h 8, *n* (%)	17 (35)	8 (33)	9 (37)	NS
Ara h 9, *n* (%)	28 (58)	17 (71)	11 (46)	NS
Hazelnut, *n* (%)	43 (90)	21 (87)	22 (92)	NS
Walnut, *n* (%)	34 (71)	15 (62)	19 (79)	NS
Rice, *n* (%)	28 (58)	13 (54)	15 (62)	NS
Wheat, *n* (%)	35 (73)	16 (67)	9 (37)	<0.05
Soy, *n* (%)	33 (69)	13 (54)	20 (83)	<0.05
Apple, *n* (%)	31 (65)	14 (58)	17 (71)	NS

Probability (*P*) values of less than 0.05 were considered significant. NA: not applicable; NS: not significant.

**Table 2:** Rates of sensitization against one of the five peanuts' molecular components (rAra h 1, 2, 3, 8, and 9) in a population of 48 children and adolescents (median age: 8 years; range: 2–16) with documented peanut sensitization (sIgE > 1 kUA/L). Patients sensitized against one allergenic component were defined as “monosensitized,” whereas those sensitized to more than one of the five peanuts' allergens were defined as “polysensitized.” Patients were categorized according to the tolerance status against peanuts.

	Study population (*n* = 48)	Tolerant patients (*n* = 24)	Avoiders (*n* = 24)	*P* values Tolerant versus avoiders
Monosensitized patients, *n* (%)	26 (54)	15 (62)	11 (46)	NS
Polysensitized patients, *n* (%)	20 (42)	8 (33)	12 (50)	NS
Two allergens, *n* (%)	14 (29)	7 (29)	7 (29)	NS
Three allergens, *n* (%)	4 (8)	1 (4)	3 (12)	NS
Four allergens, *n* (%)	1 (2)	0	1 (4)	NS
Five allergens, *n* (%)	1 (2)	0	1 (4)	NS
None of the 5 allergens, *n* (%)	2 (4)	1 (4)	1 (4)	NS

Probability (*P*) values of less than 0.05 were considered significant. NS: not significant.
